# Glucocorticoid receptor gene polymorphisms and disease activity during pregnancy and the postpartum period in rheumatoid arthritis

**DOI:** 10.1186/ar4014

**Published:** 2012-08-13

**Authors:** Rogier AM Quax, Yaël A de Man, Jan W Koper, Elisabeth FC van Rossum, Sten P Willemsen, Steven WJ Lamberts, Johanna MW Hazes, Radboud JEM Dolhain, Richard A Feelders

**Affiliations:** 1 Department of Internal Medicine, Erasmus MC, University Medical Center, 's-Gravendijkwal 230, Rotterdam, 3015 CE, The Netherlands; 2 Department of Rheumatology, Erasmus MC, University Medical Center, 's-Gravendijkwal 230, Rotterdam, 3015 CE, The Netherlands; 3 Department of Epidemiology and Biostatistics, Erasmus MC, University Medical Center, 's-Gravendijkwal 230, Rotterdam, 3015 CE, The Netherlands

## Abstract

**Introduction:**

The mechanism underlying the spontaneous improvement of rheumatoid arthritis (RA) during pregnancy and the subsequent postpartum flare is incompletely understood, and the disease course varies widely between pregnant RA patients. In pregnancy, total and free levels of cortisol increase gradually, followed by a postpartum decrease to prepregnancy values. The *glucocorticoid receptor (GR) *polymorphisms *Bcl*I and N363S are associated with relatively increased glucocorticoid (GC) sensitivity, whereas the 9β and ER22/23EK polymorphisms of the *GR *gene are associated with a relatively decreased GC sensitivity. We examined the relation between the presence of these *GR *polymorphisms and level of disease activity and disease course of RA during pregnancy and postpartum.

**Methods:**

We studied 147 participants of the PARA study (Pregnancy-Induced Amelioration of Rheumatoid Arthritis study), a prospective study investigating the natural improvement during pregnancy and the postpartum flare in women with RA. Patients were visited, preferably before pregnancy, at each trimester and at three postpartum time points. On all occasions, disease activity was scored by using DAS28. All patients were genotyped for the *GR *polymorphisms *Bcl*I, N363S, 9β, and ER22/23EK and divided in groups harboring either polymorphisms conferring increased GC sensitivity (*Bcl*I and N363S; GC-S patients) or polymorphisms conferring decreased GC sensitivity (9β or 9β + ER22/23EK; GC-I patients). Data were analyzed by using a mixed linear model, comparing GC-S patients with GC-I patients with respect to improvement during pregnancy and the postpartum flare. The cumulative disease activity was calculated by using time-integrated values (area under the curve, AUC) of DAS28 in GC-I patients versus GC-S patients. Separate analyses were performed according to the state of GC use.

**Results:**

GC-S patients treated with GC had a significantly lower AUC of DAS28 in the postpartum period than did GC-I patients. This difference was not observed in patients who were not treated with GCs. During pregnancy, GC-S and GC-I patients had comparable levels of disease activity and course of disease.

**Conclusions:**

Differences in relative GC sensitivity, as determined by *GR *polymorphisms, are associated with the level of disease activity in the postpartum period in GC-treated patients, but they do not seem to influence the course of the disease *per se*.

## Introduction

Rheumatoid arthritis (RA) is a systemic inflammatory disorder characterized by chronic synovitis leading to joint destruction. During pregnancy, spontaneous reduction of disease activity in RA is common, a phenomenon that is also observed in other autoimmune disorders [1-5]. After birth, however, RA deteriorates in the majority of women [3,4,6]. Pregnancy is supposed to have immunomodulatory effects, but the exact mechanisms underlying the spontaneous amelioration during pregnancy and the subsequent postpartum flare have still not been elucidated. Several hypotheses have, however, been put forward, including the beneficial effect of maternal-fetal HLA-incompatibility [7,8] and of increased galactosylation of immunoglobulin G [9-11]. Shifts in T-cell cytokine secretion profiles also have been proposed as a potential mechanism underlying the improvement of RA during pregnancy and the postpartum deterioration [12-15].

In healthy pregnancy, total and free levels of cortisol increase progressively, reaching a peak in the second and third trimesters [16-18]. The improvement in RA starts in the first trimester, and almost half of patients have at least low disease activity (DAS28 <3.2) in the third trimester [4]. Nevertheless, prospectively studied cohorts of pregnant RA patients concurrently evaluating reduction of disease activity with accompanying (free) cortisol levels on an individual basis are lacking. It is known from daily clinical practice, however, that interindividual differences in the degree of pregnancy-induced remission and the postpartum deterioration do exist, with some women reaching complete remission during pregnancy, whereas others have persistent active disease. This discrepancy was already noticed in two early case series in which a cortisol metabolite (that is, 17-hydroxycorticosteroid (17-OHCS)) was measured in pregnant RA women. High levels of 17-OHCS related to improvement of disease activity in only a subset of patients [19,20]. This variation in clinical responses does not depend solely on the absolute levels of cortisol but might also be explained by differences in individual GC sensitivity.

In the healthy population, a considerable variation in GC sensitivity has been demonstrated by low-dose (0.25 mg) dexamethasone suppression tests and functional *in vitro *assays [21,22]. In diseased states, these differences in GC sensitivity are reflected by a wide spectrum of GC therapy efficacy, which may partly be explained by four functional single nucleotide polymorphisms (SNPs) in the *glucocorticoid receptor (GR) *gene. The minor alleles of the polymorphisms N363S (rs6195) and *Bcl*I (rs41423247) are associated with a relative hypersensitivity to GC, whereas the ER22/23EK (rs6189 and rs6190) and 9β (rs6198) SNPs are associated with a relatively decreased GC sensitivity [23]. Previously, we demonstrated that carriers of the ER22/23EK variant more often had erosive disease and more frequently needed tumor necrosis factor-alpha (TNF-α) blocking therapy [24]. Similarly, these *GR *polymorphisms could explain differences in disease course during pregnancy and postpartum in RA.

Therefore, the aim of our study was to investigate the association between *GR *gene polymorphisms and level of disease activity and disease course during pregnancy and in the postpartum period in RA patients.

## Materials and methods

### Patients

All patients were participants of the PARA study (Pregnancy-Induced Amelioration of Rheumatoid Arthritis study), a nationwide prospective study investigating the natural improvement of RA during pregnancy and the postpartum flare [4]. If possible, patients were visited before conception. Patients were visited at their home address at each trimester and at 6 weeks, 12 weeks, and 26 weeks after delivery. In the present study, women who had a miscarriage were excluded from further analysis, and no woman was included twice.

### Data collection

Trained research nurses or physicians examined all patients by using a standardized 28-joint count for swelling and pain. Disease activity was calculated by using the disease activity score (DAS28) with three variables (swollen joint count, tender joint count and C-reactive protein (CRP) level) [25], because this variant of the DAS has been shown to reflect disease activity most reliably during pregnancy [26]. Current medication use at each visit was recorded. All mothers provided information on breastfeeding, because this may interfere with resumption of methotrexate (MTX) therapy after delivery.

Improvement of disease activity during pregnancy was defined according to the EULAR criteria as responders (moderate and good response combined) versus nonresponders and could, in accordance with the EULAR criteria, be applied only to those patients with a baseline DAS28 ≥3.2 at the first trimester (*n *= 71) [25]. The "reversed" EULAR criteria were used to define a very early flare (deterioration between the visits at the third trimester and at 6 weeks postpartum), early flare (deterioration between the visits at 6 weeks and at 3 months postpartum), and late flare (deterioration between the visits at 6 weeks and at 6 months postpartum), as described previously [4], with minor modifications (see Additional file 1, Table S1).

### Glucocorticoid-receptor polymorphisms

All patients were genotyped for four functional polymorphisms of the *GR *gene (ER22/23EK, rs6189 and rs6190; N363S, rs6195; *Bcl*I, rs41423247 and 9β, rs6198), by using DNA extracted from samples of peripheral venous blood. Genotyping was performed by using Taqman allelic discrimination assays (Applied Biosystems, Nieuwerkerk a/d IJssel, The Netherlands), following protocols described by the supplier. Results were analyzed by using the sequence detection system 2.2 software (Applied Biosystems).

### Data and statistical analysis

Mann-Whitney *U *tests and χ^2 ^tests were used to determine differences in baseline characteristics.

We estimated DAS28 in patients who used GCs versus patients who did not use GCs by using a linear mixed model (LMM). With this model, we compared the area under the curve (AUC) of DAS28 in the two groups on the whole trajectory, during pregnancy, and in the postpartum period. We used the DAS28 score as the response, and Time and the Use of glucocorticoid × Time interaction as covariates. Time is used as a categoric variable denoting one of the seven measurement occasions. Similarly, we then estimated separate linear mixed models for each individual polymorphism, by using Time and the interaction of Time × Carriage of minor alleles as covariates. Because of the low frequencies of the N363S (4.1%) and the ER22/23EK (7.5%) carriers, no AUC of DAS28 could be calculated for these models. Subjects were therefore further analyzed as carriers of a polymorphism associated with increased sensitivity for GCs (*Bcl*I and/or N363S, referred to as the GC-S group) versus carriers of a polymorphism associated with reduced sensitivity to GCs (9β or 9β + ER22/23EK, referred to as the GC-I group). Patients who were heterozygous for both the *Bcl*I and 9β polymorphisms or the N363S and 9β variants were excluded from the GC-S/GC-I groups. In this final model, we again tested whether the average DAS28 was equal between the GC-S and GC-I groups on the whole profile, during pregnancy and postpartum. In all models, we used a person-specific intercept and assumed that the residual covariance structure was autoregressive heteroskedastic.

χ^2 ^analysis was applied to compare rates of response during pregnancy and the presence of a very early, early, or late flare. All previously mentioned analyses were performed in patients who used GCs and in patients who did not use GCs separately. Patients were designated as GC-users when patients used GCs during pregnancy and used GCs at the time of at least two of three postpartum visits. No correction for multiple comparisons was applied. Differences in the median daily dosage of prednisone given during pregnancy and postpartum were calculated by using the Mann-Whitney test. Statistical analysis was performed by using the SPSS version 17.0 and SAS version 9.2. We considered differences statistically significant if *P *≤ 0.05 (two-sided).

### Ethical approval

All subjects signed informed consent, and the study was approved by the medical ethics committee of the Erasmus Medical Center. This study is in compliance with the Declaration of Helsinki.

## Results

### Baseline characteristics

In total, 147 patients participating in the PARA study were enrolled in the current study. More than 60% of patients had active disease in the first trimester of their pregnancy, and all women fulfilled the ACR 1987 revised criteria for RA (Table 1).

**Table 1:** Patient characteristics

	*n *= 147
Age at delivery in years, mean (SD)	32.4 (3.8)
Disease duration in years, median (range)	5.5 (0.1-28.4)
Gestational age at delivery in weeks, mean (SD)	39.3 (1.9)
Anti-CCP positive, *n *(%)	87 (59.2)
Rheumatoid factor (IgM) positive, *n *(%)	110 (74.8)
Presence of erosions, *n *(%)	105 (71.4)
Number of DMARDs before conception, median (range)	2 (0-6)
Breastfeeding (6 weeks postpartum), *n *(%)	60 (40.8)
DAS28-CRP3 ≥3.2 in first trimester, *n *(%)^a^	71 (61.7)
Moderate/good response during pregnancy, *n *(%)^b^	32 (45.1)
Very early flare, N (%)^c^	29 (21.5)
Early flare, *n *(%)^d^	29 (22.0)
Late flare, *n *(%)^e^	37 (30.1)

^a^In 115 patients, DAS28 in the first trimester was available. ^b^According to EULAR response criteria, DAS28 ≥3.2 in the first trimester is required. Data were available in ^c^135 , ^d^132, and ^e^123 patients, respectively, according to reversed EULAR response criteria. Very early flare, deterioration between the visit at the third trimester and at 6 weeks postpartum; early flare, deterioration between visits at 6 weeks and at 3 months postpartum; late flare, deterioration between visits at 6 weeks and at 6 months postpartum; anti-CCP, anti-cyclic citrullinated protein. DMARDs, Disease-modifying antirheumatic drugs, including prednisone.

As shown previously, sulfasalazine and prednisone were the most frequently used treatment regimens during pregnancy [4]. Approximately 40% of patients did not use any antirheumatic drug (see Additional file 2, Table S2). Disease activity scores were available in 69, 115, 133, 142, 140, 137, and 131 women at the seven different study visits before conception, during pregnancy, and postpartum, respectively.

**Figure 1:**
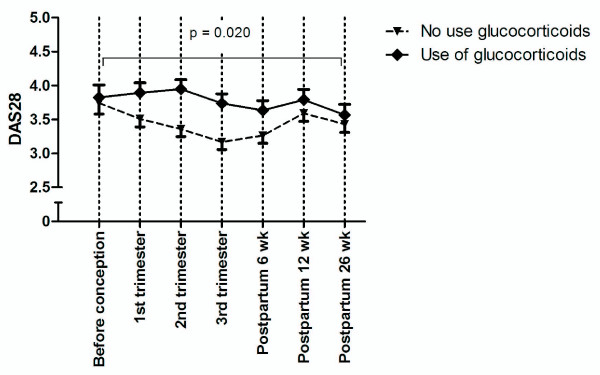
**Disease activity (DAS28 ± SEM) among pregnant women with (*n *= 57) and without (*n *= 90) use of glucocorticoids**.

In general, patients treated with GCs (*n *= 57) had significantly higher disease activity than did patients not treated with GCs (*n *= 90; Figure 1). Patients who used GCs had a significantly shorter duration of gestation and had erosions more frequently (Table 2). Analyses were therefore performed separately according to the state of GC use.

**Table 2:** Patient characteristics stratified according to use of glucocorticoids

	Use of GCs (*n *= 57)	No use of GCs (*n *= 90)
Age at delivery in years, mean (SD)	33.15 (3.80)	31.93 (3.67)
Disease duration in years, median (range)	6.07 (0.22-28.57)	5.18 (0.14-28.54)
Gestational age at delivery in weeks, mean (SD)	38.44 (2.30)	39.85 (1.27)^f^
Anti-CCP positive, *n *(%)	39 (68.4)	51 (56.7)
Rheumatoid factor (IgM) positive, *n *(%)	47 (82.5)	63 (70.0)
Presence of erosions, *n *(%)	49 (86)	56 (62.2)^f^
Dosage of prednisone (mg/day), median (range)	7.5 (2.5-20)	-
Number of DMARDs before conception, median (range)	2 (0-5)	1 (0-4)^f^
Breastfeeding (6 weeks postpartum), *n *(%)	13 (22.8)	47 (52.2)^f^
DAS28 ≥3.2 in first trimester, *n *(%)^a^	33 (70.2)	38 (55.9)
Moderate/good response during pregnancy, *n *(%)^b^	15 (45.5)	17 (43.6)
Very early flare, *n *(%)^c^	9 (17.3)	18 (21.7)
Early flare, *n *(%)^d^	9 (18.0)	20 (24.4)
Late flare, *n *(%)^e^	10 (21.3)	27 (35.5)

^a^In 115 patients, DAS28 in the first trimester was available. ^b^According to EULAR response criteria, DAS28 ≥3.2 in the first trimester is required, *n *= 71 of 115. ^c^Data were available in 135 patients, ^d^132 patients, and ^e^123 patients, according to reversed EULAR response criteria. ^f^*P *< 0.05 as compared with patients using GCs. Anti-CCP, anti-cyclic citrullinated protein.

DMARDs, Disease-modifying antirheumatic drugs, excluding prednisone.

### Glucocorticoid receptor polymorphisms and disease course during gestation and postpartum

We found 84 (57.1%) patients who were heterozygous or homozygous carriers of the *Bcl*I polymorphism. The 9β polymorphism was present in 48 (32.7%) patients.

**Figure 2:**
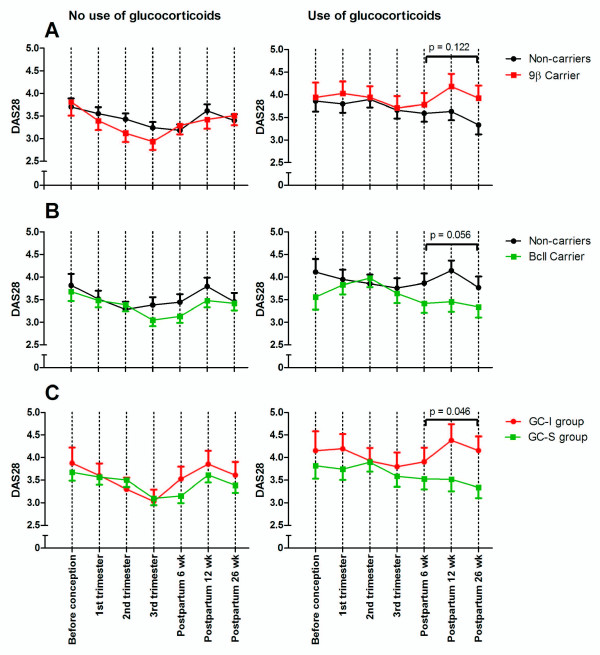
**Disease activity according to carriage of *GR *polymorphisms**. **(A) **Disease activity in carriers of 9β (*n *= 29) versus noncarriers (*n *= 61) in patients not using glucocorticoids (GCs) (left panel). Of patients using GCs, 19 were carriers of the 9β polymorphism, and 37 were noncarriers (right panel). In one patient, the 9β-genotype could not be determined. **(B) **Disease activity in carriers of *Bcl*I (*n *= 55) versus noncarriers (*n *= 34) in patients not using GCs (left panel). In one patient, the *Bcl*I-genotype could not be determined. Of patients using GCs, 29 were carriers of the *Bcl*I polymorphism, and 28 were WT carriers (right panel). **(C) **Disease activity in carriers of polymorphisms conferring increased GC sensitivity (*n *= 44; GC-S group) versus patients carrying polymorphisms conferring decreased GC sensitivity (*n *= 15; GC-I group) not using GC (left panel). Of patients using GC, 24 were in the GC-S group, and 13, in the GC-I group (right panel). Disease activity is presented as DAS28 ± SEM.

Analysis of the level of disease activity in carriers versus noncarriers of these polymorphisms showed that 9β carriers did not differ significantly in AUC of DAS28 compared with noncarriers (Figure 2A). *Bcl*I carriers treated with GC had a near-significant lower AUC of DAS28 postpartum compared with noncarriers (*P *= 0.056; Figure 2B, right panel). No differences in the AUCs of DAS28 postpartum were observed in non-GCtreated patients.

Nineteen (12.9%) patients were heterozygous carriers of both the *Bcl*I and 9β polymorphisms or the N363S and 9β variants. These patients were excluded in the final analysis to allow an appropriate comparison between patients carrying a polymorphism associated with increased sensitivity to GCs (*Bcl*I and/or N363S, GC-S group) and patients harboring a genetic variant associated with reduced sensitivity to GCs (9β or 9β + ER22/23EK, GC-I group). The results of this analysis, shown in Figure 2C, indicate that GC treated patients in the GC-I group had a significantly higher AUC of DAS28 in the whole postpartum period (that is, up to 26 weeks), than did patients in the GC-S group (*P *= 0.046). In patients not treated with GCs, these differences did not exist.

The AUC of DAS28 during pregnancy, the course of the disease, EULAR response during pregnancy, and the presence of a very early flare, early flare, or late flare with reversed EULAR response criteria, were not associated with any *GR *genotype, although the DAS28 was lower in the GC-S group than in the GC-I group at all time points in GC treated patients (Figure 2C).

The *GR *genotypes were equally distributed among GC users and non-GC users. The clinical characteristics between GC-S and GC-I patients, stratified according to the use of GCs, did not differ, except for the more frequent use of nonsteroidal antiinflammatory drugs (NSAIDs) in the GC-I group (*P *= 0.01; Table 3). The median daily dosage of prednisone given during pregnancy, taking the highest dosage needed at any time during pregnancy, tended to be higher in GC-I patients (8.75 mg daily versus 6.25 mg daily; *P *= 0.157). GC-S patients could more frequently reduce the daily needed GC dose during pregnancy than could the GC-I patients, possibly reflecting higher GC sensitivity to the pregnancy-related increase in cortisol in GC-S patients, although this was not statistically significant (*n *= 7, 29.2% versus *n *= 1, 7.7%; *P *= 0.130). In the postpartum period, prednisone daily dosages did not differ between GC-S and GC-I patients.

**Table 3:** Clinical characteristics of patients in the GC-S and GC-I groups according to the use of glucocorticoids

	Use of GCs		No use of GCs	
	
	GC-S (*n *= 24)	GC-I (*n *= 13)	GC-S (*n *= 44)	GC-I (*n *= 15)
Age at delivery in years, mean (SD)	34.1 (3.1)	34.1 (3.6)	31.6 (3.9)	31.2 (3.0)
Disease duration in years, median (range)	4.6 (0.2-28.6)	6.8 (1.0-22.7)	5.3 (0.1-28.5)	2.4 (0.2-28.4)
Gestational age at delivery in weeks, mean (SD)	39.0 (1.9)^A^	37.4 (2.2)	39.8 (1.3)	39.9 (1.3)
Anti-CCP positive, *n *(%)	16 (66.7)	8 (61.5)	24 (54.5)	6 (40%)
Rheumatoid factor (IgM) positive, *n *(%)	17 (70.8)	10 (76.9)	31 (70.5)	9 (60.0)
Presence of erosions, *n *(%)	22 (91.7)	13 (100)	28 (63.6)	8 (53.3)
Dosage of prednisone (pregnancy; mg/day), median (range)	6.25 (2.5-15)	8.75 (5-20)	-	-
Dosage of prednisone (postpartum; mg/day), median (range)	8.75 (2.5-15)	10.0 (5-15)	-	-
Number of DMARDs before conception, median (range)	2 (0-4)	2 (1-5)	2 (0-4)	2 (0-3)
Moderate/good response during pregnancy, *n *(%)	5/11 (45.5)	4/9 (44.4)	8/16 (50)	5/11 (45.5)
Very early flare, *N/N*_total _(%)	5/21 (23.8)	3/13 (23.1)	10/41 (24.4)	5/13 (38.5)
Early flare, *N/N*_total _(%)	4/21 (19.0)	2/12 (16.7)	12/40 (30.0)	3/13 (23.1)
Late flare, *N/N*_total _(%)	4/19 (21.1)	4/13 (30.8)	15/37 (40.5)	4/12 (33.3)
Breastfeeding (6 weeks postpartum), *n *(%)	8 (33.3)	1 (7.7)	20 (45.5)	8 (53.3)
Use of NSAIDs at 6 months postpartum,^a ^*N/N*_total _(%)	7/22 (31.8)^b^	10/13 (76.9)	13/40 (32.5)	6/13 (46.2)
Use of MTX at 6 months postpartum,^a ^*N/N*_total _(%)	11/22 (50.0)	9/13 (69.2)	10/40 (25.0)	5/13 (38.5)
Use of sulfasalazine at 6 months postpartum,^a ^*N/N*_total _(%)	6/22 (27.3)	2/13 (15.4)	17/40 (42.5)	6/13 (46.2)
Use of anti-TNF-α at 6 months postpartum,^a ^*N/N*_total _(%)	3/22 (13.6)	3/13 (23.1)	2/40 (5.0)	0/13 (0)

Data concerning response during pregnancy, very early flare, early flare, and late flare were present in 47, 88, 86, and 81 patients, respectively. ^a^Available in 88 patients. ^b^*P *< 0.05 compared with GC-I, use of GC. Anti-CCP, anti-cyclic citrullinated protein; TNF-α, tumor necrosis factor-alpha.

## Discussion

In this nationwide prospective study including 147 pregnant RA patients, we examined for the first time whether *GR *polymorphisms that modulate GC sensitivity are associated with the level of disease activity and disease course during pregnancy and the postpartum period. We show that GC treated patients in the GC-S group (that is, those with the *Bcl*I or N363S or both polymorphisms, associated with relatively increased GC sensitivity) have a significantly lower disease activity in the postpartum period than do patients in the GC-I group (9β or 9β + ER22/23EK, associated with relatively decreased GC sensitivity), as measured by the AUC of the DAS28. In patients not treated with GC, the level of disease activity and disease course during pregnancy or in the postpartum period does not seem to be influenced by differences in *GR *genotype.

Gestational-induced remission of RA has been recognized for a long time [27] and may in part be attributed to the increase in cortisol production that in turn enhances endogenous immunosuppression. Pregnancy is indeed considered to be a natural variant of hypercortisolism [28,29] and serum (free) cortisol, urinary free cortisol, salivary cortisol, and cortisol content in hair all have been demonstrated to increase progressively during gestation, followed by a rapid postpartum decrease in cortisol levels [17,18,30-37].

Apart from cortisol availability, the ultimate biologic effects of GCs also depend on GC sensitivity, which is modulated by *GR *polymorphisms [23].

Based on the course of cortisol levels during pregnancy and after delivery, we hypothesized that differences in glucocorticoid sensitivity might in part explain why the beneficial effect of pregnancy on RA disease activity does not occur in all RA patients.

Polymorphisms of the *GR *gene have been demonstrated to influence disease course in several inflammatory disorders, including Graves ophthalmopathy [38], Crohn disease [39], and multiple sclerosis [40]. We recently demonstrated that the minor alleles of *Bcl*I and 9β were associated, respectively, with decreased and increased susceptibility to develop RA. In addition, ER22/23EK carriers had a worse disease phenotype and needed more frequent TNF-α blocking therapy [24]. We extend these data by demonstrating higher levels of disease activity in the postpartum period in GC treated patients in the GC-I group, despite the more frequent use of NSAIDs.

Interestingly, the differences in disease activity between carriers of GC-sensitive and GC-resistant polymorphisms were observed only in women treated with GCs. The GC treated patients involve a subgroup of women with high disease activity, as reflected by observed higher DAS28. Our observations may imply that in the postpartum phase, when endogenous cortisol levels decrease, patients with polymorphisms associated with increased GC sensitivity have more benefit from GC therapy. Therefore, in states of relative glucocorticoid deficiency, differences in GC sensitivity due to genetic variability may in part determine variations in disease activity. Conversely, in patients with low disease activity, as characterized by the absence of glucocorticoid therapy in our cohort, endogenous levels of cortisol apparently can prevent uncontrolled inflammatory processes independent of genetic variations of the *GR *gene, although we did not measure cortisol levels in our patients.

This concept of a "relative glucocorticoid deficiency" might also explain why the observed variation in disease activity seems to be restricted to the postpartum period, because Magiakou and co-workers [41] showed that hypothalamic CRH secretion in healthy pregnant women is transiently suppressed at 3 and 6 weeks, recovering only at 12 weeks postpartum. This suppression of the hypothalamic-pituitary-adrenal (HPA) axis in the postpartum period, which could be even more pronounced in RA in which a preexisting blunted HPA axis is described in nonpregnant states [42], might even further attenuate the ability of the HPA axis to produce sufficient levels of cortisol.

The clinical relevance of this blunted HPA axis in the first 3 months after childbirth is illustrated by a higher incidence or exacerbation of several autoimmune diseases, including postpartum depression, autoimmune thyroid disease, and rheumatoid arthritis itself [41,43-46]. The lack of differences between GC-I and GC-S patients in disease activity during pregnancy could also be explained by altering levels of glucocorticoid sensitivity, as was suggested by Majzoub and co-workers [47,48]. Alternatively, patients in the GC-I group tended to need higher daily dosages of GCs during pregnancy, which could have masked a higher level of disease activity in this subgroup of patients. Although we focused on glucocorticoids, absolute levels of estrogens and progesterone also increase progressively during gestation. Both estrogens and progesterone possess antiinflammatory properties and are therefore likely to have substantially influenced the disease course [49]. Similar to differences in GC sensitivity, one could speculate that variation in sensitivity to the immunosuppressing effects of estrogens and progesterone might also contribute to the wide clinical spectrum of changes in disease activity observed in pregnancy and after delivery in RA.

Interestingly, the difference in disease activity between GC-I and GC-S patients persisted during the entire postpartum follow-up period (that is, up to 26 weeks). Future studies should examine at which time points disease activity patterns of both groups converge to prepregnancy levels.

It should be noted that our study also has some limitations. First, genetic-association studies usually require larger numbers of patients. Although this is the largest prospectively studied cohort of pregnant RA patients, additional studies are needed to validate our findings. Second, the presented data are based on Caucasian patients only, who may differ from patients from other geographic areas with different genetic and environmental backgrounds. Third, parameters of HPA axis activity, not measured in this study, could have provided additional information in the non-GC treated patients.

Although the pattern of cortisol levels in pregnancy and after delivery has been extensively documented [17,18,30-37], large prospective studies evaluating cortisol levels along with clinical responses during pregnancy and postpartum in RA are currently lacking. Together with new insights in the past two decades supporting a blunted HPA axis in RA, this justifies renewed interest in the precise role of GC in pregnant RA patients and the course of disease [42,50]. In this context, long-term indices of HPA axis activity, as measured by means of cortisol in hair, together with dynamic functional assays to assess GC sensitivity (that is, GR number, affinity of the GR receptor, and GR-mediated gene transcription) are promising techniques to unravel further the role of GCs and the precise contribution to pregnancy-associated alterations in disease activity in RA.

## Conclusions

We demonstrate that differences in GC sensitivity, as determined by *GR *polymorphisms, might influence the level of disease activity in the postpartum period in GC treated women. The course of the disease itself does not seem to be associated with polymorphisms of the *GR*. In the light of the relatively small numbers of patients in each genotype group, however, our data should be regarded as an interesting new hypothesis possibly adding to the elucidation of the multifactorial mechanisms underlying pregnancy-induced amelioration and the postpartum flare, but the data do not necessarily prove the genetic association. Therefore, future (larger) studies should validate our hypothesis and examine both parameters of glucocorticoid availability and parameters of glucocorticoid sensitivity in relation to individual disease courses of pregnant RA patients.

## Abbreviations

AUC: area under the curve; GC: glucocorticoid; GR: glucocorticoid receptor; HPA axis: hypothalamic-pituitary-adrenal axis; LMM: linear mixed model; MTX: methotrexate; NSAIDs: nonsteroidal antiinflammatory drugs; PARA study: Pregnancy-Induced Amelioration of Rheumatoid Arthritis study; RA: rheumatoid arthritis; SNPs: single-nucleotide polymorphisms; TNF-α: tumor necrosis factor-alpha.

## Competing interests

The authors declare that they have no competing interests.

## Authors' contributions

RAMQ carried out the laboratory work and wrote the article. YAdM, JMWH, and RJEMD participated in the study design, collection of patient data, co-writing the article, and research supervision. JWK, EFCvR, SWJL, and RAF participated in co-writing the article and research supervision. SPW did the statistical analysis and participated in co-writing the article. All authors read and approved the manuscript for publication.

## Supplementary Material

Additional file 1 **Table S1. Reversed EULAR response criteria for the definition of postpartum deterioration**. This table shows the conditions for classifying patients as having no flare or a moderate or severe flare. Click here for file

Additional file 2 **Table S2. Medication use**. This table gives an overview of the different antirheumatic drugs (prednisone, NSAIDs, DMARDs, and biologicals) used by the patients at different stages of pregnancy and postpartum. Click here for file
