# Supplementary material for: Glucocorticoid receptor gene polymorphisms and disease activity during pregnancy and the postpartum period in rheumatoid arthritis
Source: Arthritis Res Ther. 2012 Aug 13;14(4):R183. doi: 10.1186/ar4014 (PMC3580579; doi:10.1186/ar4014)
Supplement: Additional file 1 — Table S1. Reversed EULAR response criteria for the definition of postpartum deterioration. This table shows the conditions for classifying patients as having no flare or a moderate or severe flare. [file ar4014-S1.DOCX]

| **Supplementary Table 1 ‘Reversed’ EULAR response criteria for the definition of deterioration postpartum.** | | | |
| --- | --- | --- | --- |
|  | ***Increase of DAS28 with*** | | |
| **DAS28 at 6*,12** or 26*** weeks postpartum** | **>1.2** | **>0.6 and ≤1.2** | **≤0.6** |
| >5.1 | Severe flare | Moderate flare | No flare |
| >3.2 and ≤5.1 | Moderate flare | Moderate flare | No flare |
| ≤3.2 | Moderate flare | No flare | No flare |
| Deterioration of disease activity is studied from trimester 3 to 6 weeks postpartum (*very early flare), 6 weeks postpartum to 12 weeks postpartum (**early flare) and from 6 weeks postpartum to 26 weeks postpartum (***late flare). No baseline disease activity is required. | | | |
