# Supplementary material for: Glucocorticoid receptor gene polymorphisms and disease activity during pregnancy and the postpartum period in rheumatoid arthritis
Source: Arthritis Res Ther. 2012 Aug 13;14(4):R183. doi: 10.1186/ar4014 (PMC3580579; doi:10.1186/ar4014)
Supplement: Additional file 2 — Table S2. Medication use. This table gives an overview of the different antirheumatic drugs (prednisone, NSAIDs, DMARDs, and biologicals) used by the patients at different stages of pregnancy and postpartum. [file ar4014-S2.DOCX]

| Supplementary Table 2. Medication use | | | | | | | |
| --- | --- | --- | --- | --- | --- | --- | --- |
| Medication | Before pregnancy  (N=69) | 1^st^ trimester  (N=115) | 2^nd^ trimester  (N=133) | 3^rd^ trimester  (N=142) | PP-1  (N=139) | PP-2  (N=137) | PP-3  (N=129) |
| Prednisone | 28 (40.6) | 45 (39.1) | 51 (38.3) | 51 (35.9) | 50 (36.0) | 47 (34.3) | 44 (34.1) |
| Sulfasalazine | 24 (34.8) | 33 (28.7) | 38 (28.6) | 40 (28.2) | 40 (28.8) | 45 (32.8) | 42 (32.6) |
| Hydroxychloroquine | 2 (2.9) | 2 (1.7) | 3 (2.3) | 2 (1.4) | 4 (2.9) | 8 (5.8) | 9 (7.0) |
| Methotrexate | 0 (0) | 0 (0) | 0 (0) | 0 (0) | 21 (15.1) | 38 (27.7) | 51 (39.5) |
| TNF-α blocking agents | 1 (1.4) | 0 (0) | 0 (0) | 0 (0) | 6 (4.3) | 12 (8.8) | 13 (10.1) |
| NSAIDs | 1 (1.4) | 7 (6.1) | 5 (3.8) | 3 (2.1) | 29 (20.9) | 57 (41.6) | 49 (38.0) |
| Other | 3 (4.3) | 1 (0.8) | 1 (0.8) | 1 (0.7) | 1 (0.7) | 4 (2.9) | 5 (3.9) |
| No medication | 23 (33.3) | 47 (40.9) | 53 (39.8) | 63 (44.4) | 46 (33.1) | 24 (17.5) | 19 (14.7) |

Values are presented as N(%). PP-1: postpartum visit after 4-6 weeks, PP-2: postpartum visit after 12 weeks, PP-3: postpartum visit after 26 weeks. The percentages presented here do not add up to 100%, as patients may use more than one antirheumatic drug. Data about use of medication were missing in 1 and 2 patients at postpartum visit 1 and 3 respectively.
